# Nonsurgical Treatment of Oral Mucocele With Intralesional Corticosteroid Injections: A Case Report

**DOI:** 10.7759/cureus.67363

**Published:** 2024-08-21

**Authors:** Gaurav S Yermalkar, N.D. Shashikiran, Namrata Gaonkar, Sachin Gugawad, Savita G Hadakar, Sonali Waghmode

**Affiliations:** 1 Department of Paedodontics and Preventive Dentistry, School of Dental Sciences, Krishna Vishwa Vidyapeeth (Deemed to be University), Karad, IND

**Keywords:** behavior management, habits, lip bumper, triamcinolone acetonide, mucocele

## Abstract

Oral mucoceles are common lesions resulting from alterations in minor salivary glands due to mucus accumulation. Deleterious habits such as lip biting, sucking, or trauma from oral appliances can result in the occurrence of mucoceles. Although conventional surgical removal has been the preferred treatment option, it is associated with drawbacks, including the risk of damaging nearby ducts and the formation of satellite lesions. A 13-year-old male patient visited the department with a sessile, nodular, and exophytic lesion on the lower lip and a persistent history of traumatic lip biting. The treatment involved the administration of intralesional injections of triamcinolone acetonide at the base of the lesion. A significant reduction was noted after the first injection, with the lesion showing complete resolution within two weeks. No recurrence was observed during the six-month follow-up period. This case highlights the effectiveness of intralesional corticosteroid injections as a non-surgical treatment option for mucoceles. This treatment modality, due to its non-invasive nature, can be particularly considered as the primary treatment choice in the pediatric population, facilitating effective behavior management.

## Introduction

Mucoceles are mucus-filled cysts that can form in various locations, such as the lacrimal sac, paranasal sinus, oral cavity, gallbladder, and appendix [1]. The term mucocele originates from Latin words for mucus and cavity [2]. Salivary gland lesions are commonly found within the oral cavity, with mucocele being the 17th most common lesion [3]. These cysts are typically soft and fluctuate on palpation while also being painless, with a high chance of recurrence [4].

Mucoceles are primarily classified into two types: extravasation and retention. The more common extravasation type of mucocele occurs due to trauma to the main duct of the salivary glands, leading to the formation of a pseudocyst. On the other hand, an obstruction of the duct of minor or accessory salivary glands can result in the formation of the retention type [5]. Clinically, mucoceles present as asymptomatic vesicles or bullae with a pink or bluish hue. The size of a mucocele can range from 1 mm to several centimeters, affecting individuals of any age and gender, most specifically between ages 10 and 20 [1]. Mucocele most commonly affects the lower labial mucosa, but it can also appear on the floor of the mouth (ranulas), tongue, cheek, and palate [1]. Typically, these cysts present as distinct, painless swellings with smooth surfaces and can vary in size from a few millimeters to several centimeters [6].

While surgery is accepted as the standard treatment for mucoceles, alternative approaches include laser irradiation, cryosurgery, micro-marsupialization, and steroid injections [7]. For pediatric patients, noninvasive treatments are preferred for better behavior management. This report discusses the minimally invasive treatment for a large, recurrent mucocele on the lower lip of a 13-year-old boy.

## Case presentation

A 13-year-old boy was referred to the Department of Pediatric and Preventive Dentistry at the School of Dental Sciences, Karad, for a swelling on his lower lip over the past four months. His parents noticed a gradual increase in the size of the swelling, with occasional episodes of its reduction followed by its recurrence. Additionally, the mother mentioned that the patient had a deleterious lip-biting habit. The patient reported a history of a mucocele that had been surgically excised six months prior; however, it had recurred due to the patient's lip-biting habit. The currently presented swelling was painless, with no reports of fever or malaise. The patient's family history was unremarkable.

**Figure 1:**
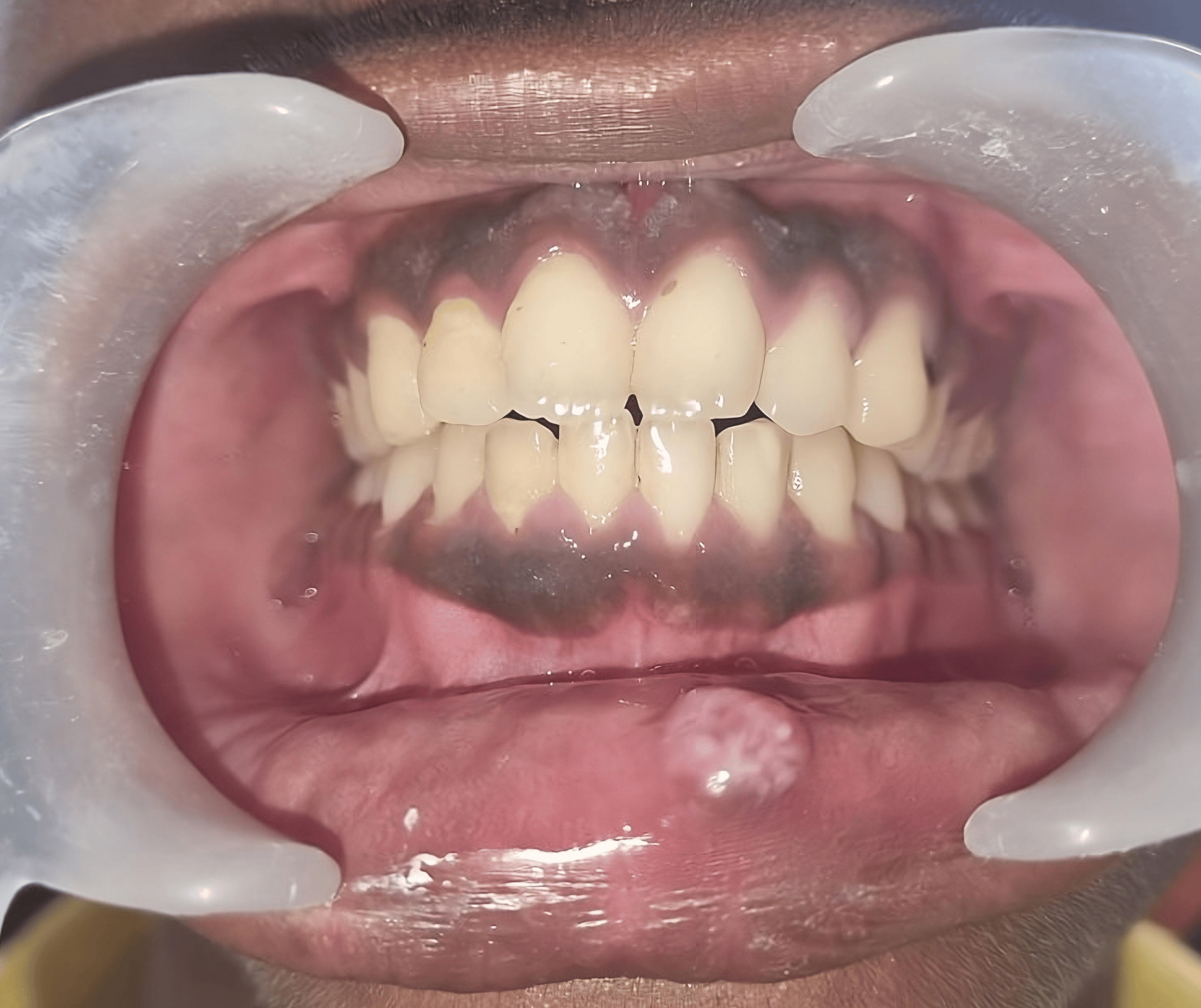
Clinical view of the lesion before treatment

An extraoral examination showed no abnormalities, while the intraoral examination revealed an exophytic, blue-colored lesion on the lower lip, which was sessile, nodular, and fluctuant in consistency. The final diagnosis of a mucocele was based on clinical observation and the patient's persistent history of lip biting (Figure 1).

**Figure 2:**
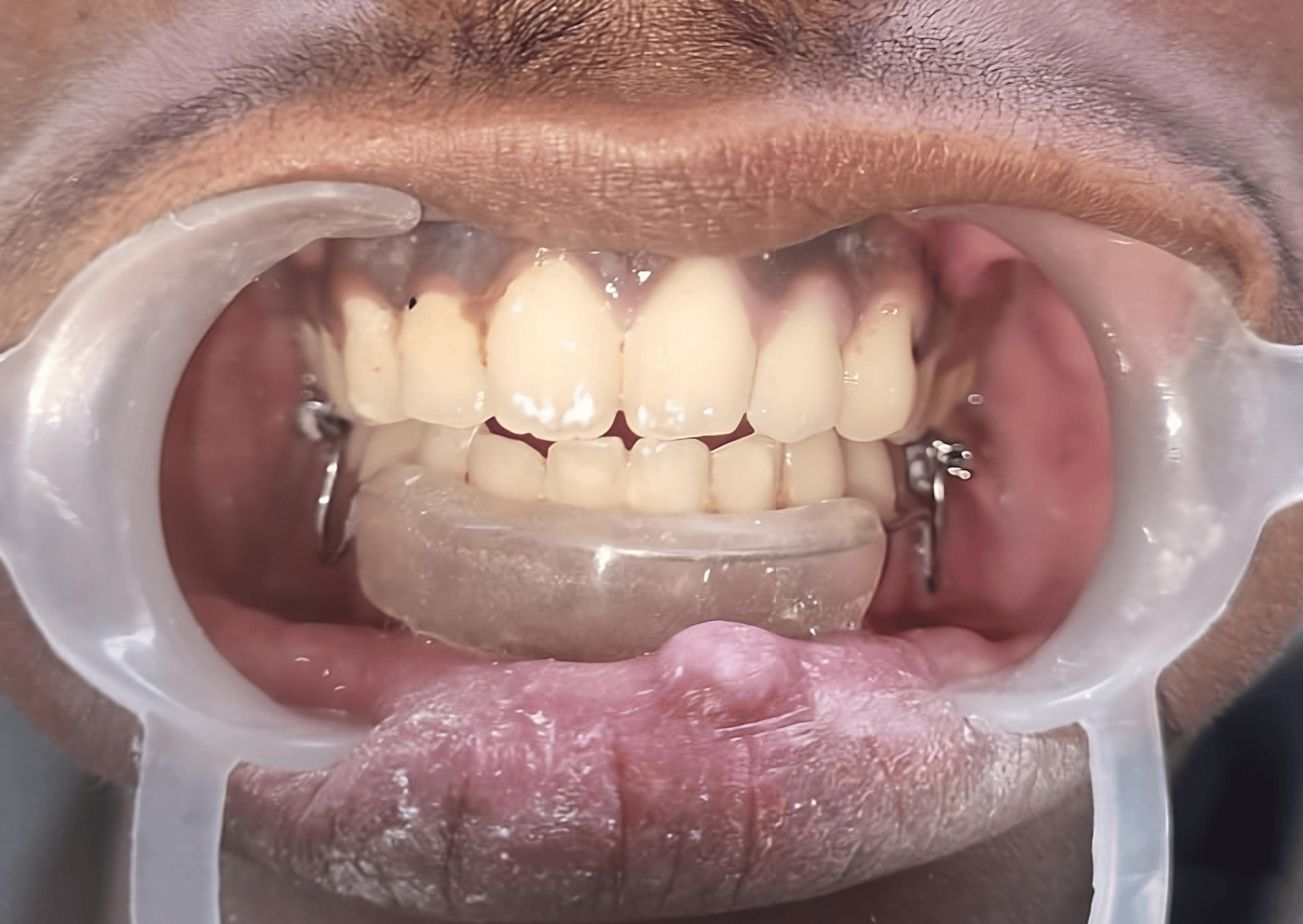
Delivery of a lip bumper

Given the invasiveness of surgical treatment and the potential for non-compliance from the patient, the clinician opted for a conservative approach of using corticosteroid injections. Written informed consent was obtained from the patient's parents. To address the lip-biting habit, a lip bumper was fabricated and provided to the patient from day one of the treatment (Figure 2).

**Figure 3:**
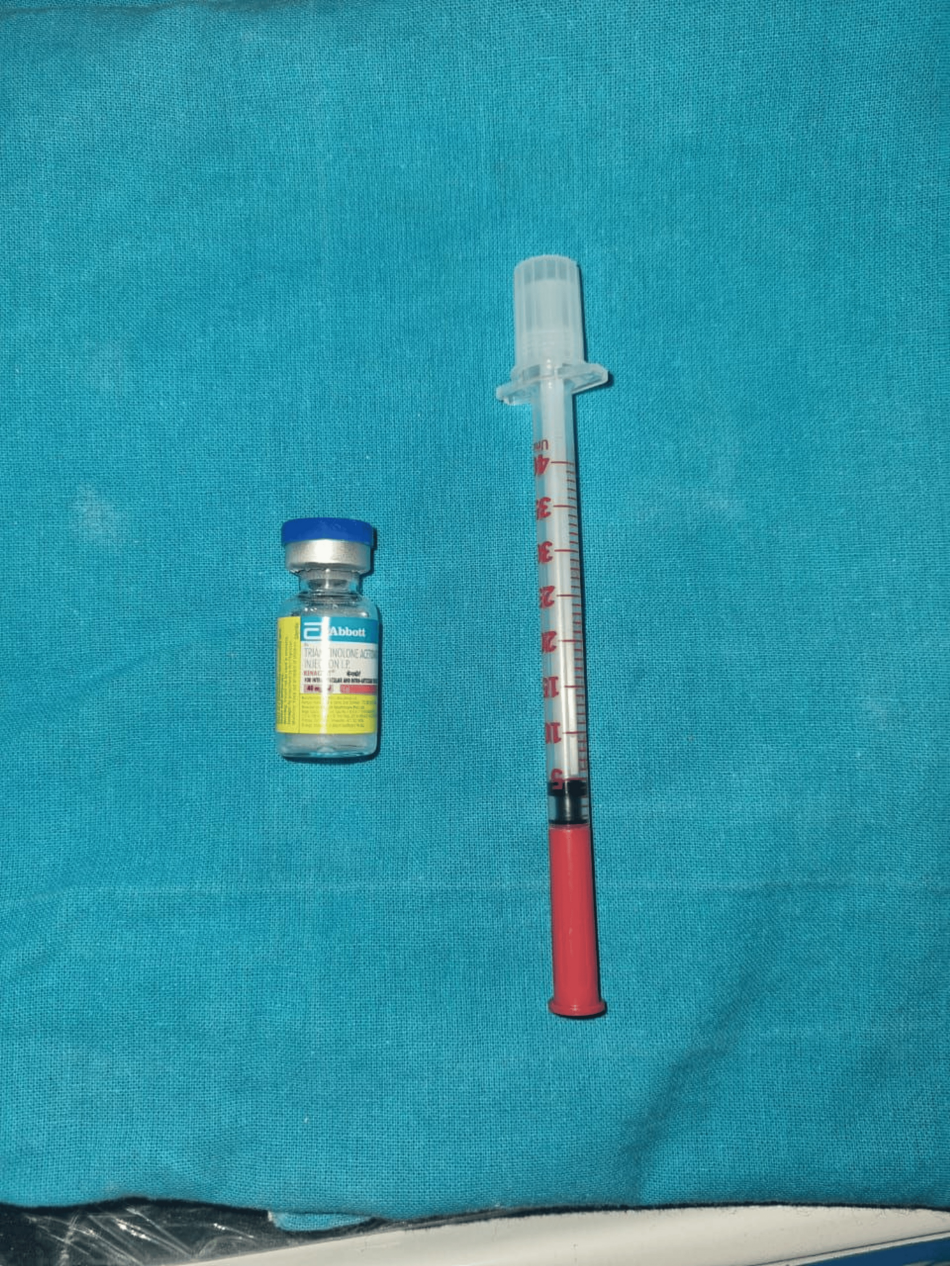
A vial of triamcinolone acetonide and an insulin syringe

**Figure 4:**
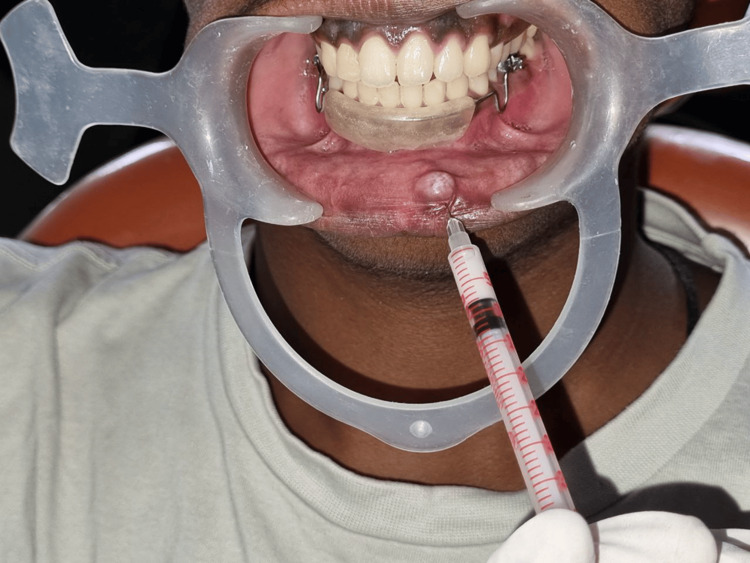
Injection of intralesional corticosteroid at the base of the mucocele

**Figure 5:**
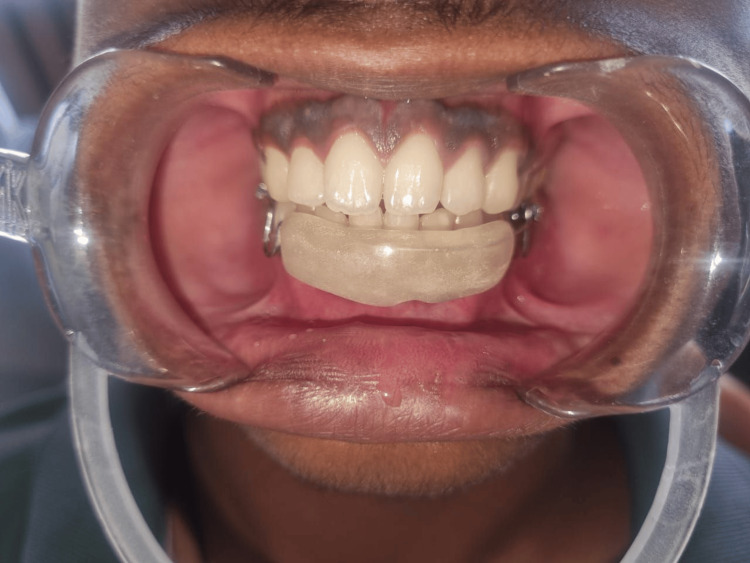
Clinical view of the lesion three weeks post-treatment

**Figure 6:**
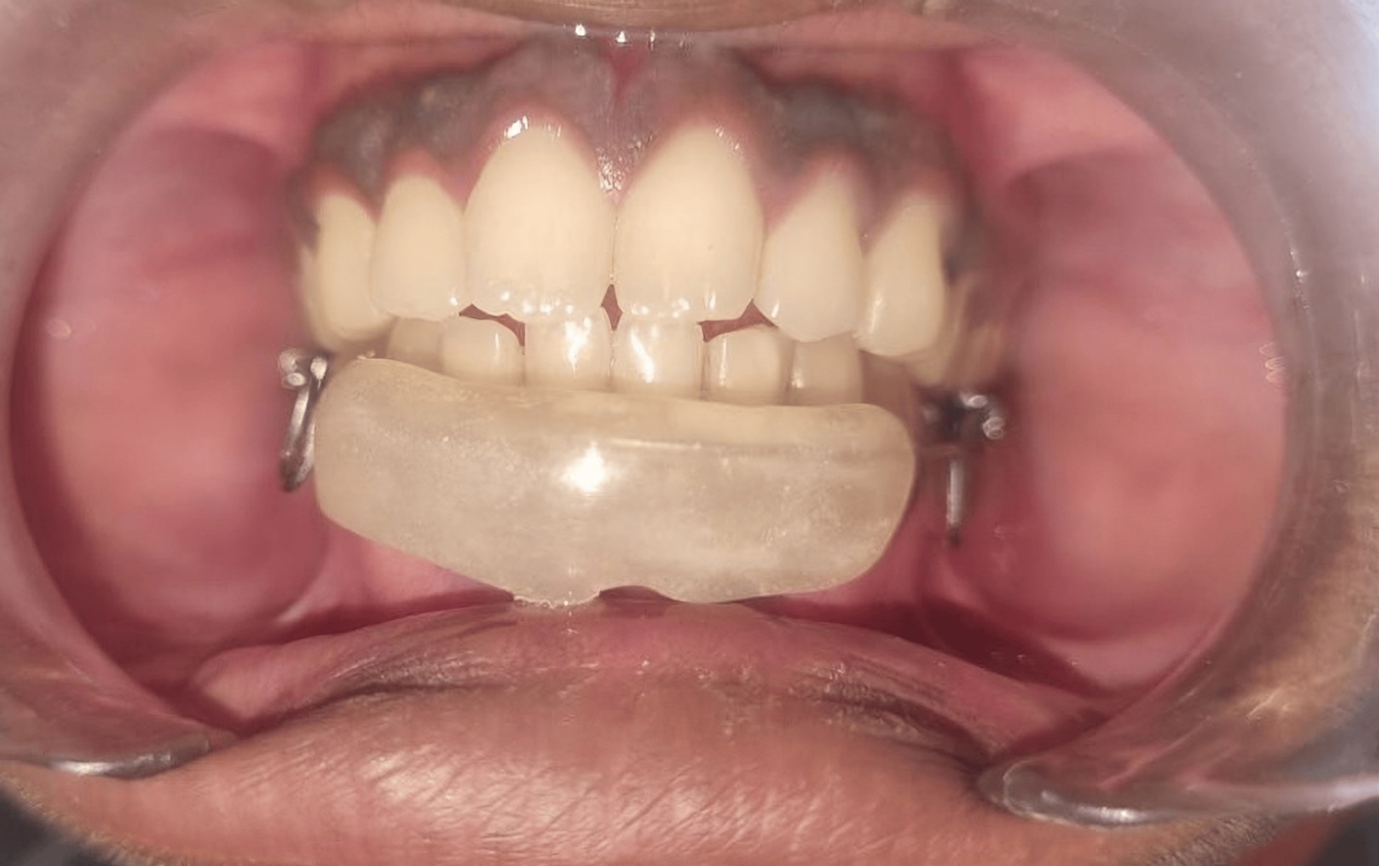
Clinical view of the lesion six months post-treatment

During the second appointment, inferior alveolar nerve anesthesia was administered, followed by two consecutive intralesional injections of 40 mg/mL triamcinolone acetonide at the base of the lesion (Figures 3, 4). The first injection consisted of 1 mL of the drug. After one week, i.e., during the third appointment, a significant reduction in the lesion's size was observed. Consequently, a second injection of 0.5 mL at the base of the lesion was given during the same appointment. The lesion completely resolved after seven days (Figure 5). The patient was monitored for six months, during which no recurrence was reported (Figure 6). The patient has subsequently been placed on a long-term follow-up to monitor his lip-biting habit.

## Discussion

Mucocele is a commonly found lesion of the oral mucosa and frequently affects the general population. An accumulation of mucous due to alterations of the minor salivary glands typically results in the formation of a mucocele [8]. These lesions are generally superficial and rarely exceed a significant diameter. Extravasation mucoceles, commonly found in minor salivary glands, occur when fluid leaks from ducts or acini into the surrounding tissue, often due to physical trauma that causes salivary secretions to infiltrate the submucosal tissue.

An accurate diagnosis is predominantly dependent on clinical evaluation and findings. The characteristic appearance of mucoceles, combined with factors such as lesion location, history of trauma, rapid onset, size fluctuations, bluish coloration, and consistency, is crucial for an accurate diagnosis [9]. Deleterious oral habits such as lip biting or sucking are identified as etiological factors for lesions such as mucoceles and irritation fibroma [10]. According to More et al., causative factors for oral mucocele include lip biting (22.41%) and trauma (5.18%) [11]. The current case report presents a patient with a history of lip biting that led to repeated minor trauma at the same site, causing the mucocele to recur even after surgical removal six months prior. To prevent recurrence, the patient's habit needed to be terminated first. Keeping this in mind, a habit-breaking appliance, a lip bumper, was delivered to the patient. As the appliance was delivered, the patient was informed that it should be worn for approximately five to six months for the effective termination of the habit [12].

Mucoceles can often be painless without causing much discomfort to the patient. However, they can impede simple oral functions such as speech, chewing, or swallowing [13]. In such cases, removal is necessary. Surgical excision, which involves removing the entire lesion and the associated salivary gland, is the conventional treatment. However, surgery may damage the adjacent minor salivary glands, potentially leading to new mucoceles, as observed in our patient. Additionally, surgery can be invasive and may cause psychological and behavioral issues in pediatric patients, necessitating sedation or general anesthesia. Alternative treatments include laser therapy, cryosurgery, micro-marsupialization, and steroid injections [9].

Managing behavior in pediatric patients is crucial, as children with oral pathologies may experience anxiety and behavioral issues. Practitioners should use minimally invasive treatments whenever possible. Among less invasive treatments, intralesional corticosteroid injections have shown good efficacy in treating recurrent mucoceles [6]. This aligns with the findings of Gholami et al., who reported the effectiveness of intralesional triamcinolone acetonide corticosteroids in the treatment of recurring mucocele [14].

Intralesional steroids offer advantages over topical ones, such as bypassing the oral mucosal barrier, reducing mucosal atrophy, and delivering higher concentrations of the drug directly to the lesion site [15]. Their effectiveness is likely due to potent vasoconstrictive and anti-inflammatory properties that reduce the inflammatory process involved in mucoceles' pathogenesis [16]. Mortazavi et al. [16] suggested combining intralesional dexamethasone with micro-marsupialization for treating mucoceles on the lower lip, while Sinha et al. [6] reported using betamethasone for non-invasive mucocele management.

The presented case report revealed that administering an intralesional injection by itself can lead to a complete resolution of the lesion while also avoiding future recurrences by using an effective habit-breaking appliance. Hence, this can be a practical and efficient method of managing cases of mucocele in the pediatric population due to its non-invasive and yet effective nature.

## Conclusions

Intralesional corticosteroid injections can be regarded as an effective treatment option for the resolution of an oral mucocele. The benefits of this non-invasive method include low cost, being aesthetically acceptable for the patient, and easy chairside reproducibility. This method is not only practical but also serves as an efficient treatment choice in the pediatric population since it can be performed in a short period while requiring minimal patient cooperation. To conclude, this treatment modality can be an effective alternative to surgical management of oral mucocele, which can directly improve behavior management in the pediatric population.
